# Highly Specific Contractions of a Single CAG/CTG Trinucleotide Repeat by TALEN in Yeast

**DOI:** 10.1371/journal.pone.0095611

**Published:** 2014-04-18

**Authors:** Guy-Franck Richard, David Viterbo, Varun Khanna, Valentine Mosbach, Lauriane Castelain, Bernard Dujon

**Affiliations:** 1 Institut Pasteur, Unité de Génétique Moléculaire des Levures, Département Génomes & Génétique, Paris, France; 2 Sorbonne Universités, UPMC Univ Paris 6, IFD, Paris, France; 3 CNRS, UMR3525, Paris, France; University of Florida, United States of America

## Abstract

Trinucleotide repeat expansions are responsible for more than two dozens severe neurological disorders in humans. A double-strand break between two short CAG/CTG trinucleotide repeats was formerly shown to induce a high frequency of repeat contractions in yeast. Here, using a dedicated TALEN, we show that induction of a double-strand break into a CAG/CTG trinucleotide repeat in heterozygous yeast diploid cells results in gene conversion of the repeat tract with near 100% efficacy, deleting the repeat tract. Induction of the same TALEN in homozygous yeast diploids leads to contractions of both repeats to a final length of 3–13 triplets, with 100% efficacy in cells that survived the double-strand breaks. Whole-genome sequencing of surviving yeast cells shows that the TALEN does not increase mutation rate. No other CAG/CTG repeat of the yeast genome showed any length alteration or mutation. No large genomic rearrangement such as aneuploidy, segmental duplication or translocation was detected. It is the first demonstration that induction of a TALEN in an eukaryotic cell leads to shortening of trinucleotide repeat tracts to lengths below pathological thresholds in humans, with 100% efficacy and very high specificity.

## Introduction

Trinucleotide repeat expansions are involved in at least two dozens dramatic neurological and developmental disorders in human [1], [2], [3], [4], [5]. A large amount of studies were devoted to understanding the mechanisms responsible for large CAG/CTG repeat expansions, using model systems as diverse as bacteria [6], [7], yeast [8], [9], [10], drosophila [11], mice [12], [13], [14], [15] or human cell lines [16], [17], [18]. Over the last 20 years or so, it was demonstrated that replication slippage, double-strand break repair, base excision repair, nucleotide excision repair, mismatch repair, basically any mechanism involving *de novo* DNA synthesis within CAG/CTG triplet repeats would favor repeat size changes (reviewed in: [1], [19], [20], [21]. However, the precise mechanism by which hundreds or thousands of triplets are added in one single human generation is still obscure.

Given that trinucleotide repeat disorders are always associated to an expansion of the repeat array, shortening the expanded array to non-pathological length should suppress the pathology. Indeed, when a large trinucleotide repeat contraction occured during transmission from father to daughter, of an expanded myotonic dystrophy allele, complete clinical examination of the daughter showed no sign of myotonic dystrophy symptoms [22]. It was previously reported that frequent expansions and contractions of a CAG/CTG repeat occured during double-strand break repair induced by a specific endonuclease such as I-Sce I [23] or HO [24], in *Saccharomyces cerevisiae* [25], [26], [27], [28]. More specifically, when an I-Sce I recognition site was inserted between two short (CAG)_5_ repeats and a double-strand break (DSB) was induced, two-thirds of the repair events led to shortening of the repeat array by single-strand annealing, even though a homologous template was available to repair the break by gene conversion [26]. This observation led us to the idea that inducing a specific DSB within a given trinucleotide repeat could lead to its shortening to non-pathological length.

Historically, the first nucleases used to induce a specific double-strand break into an eukaryotic chromosome were I-Sce I [29], [30] and HO [31]. Subsequently, homing endonucleases of the LAGLIDAGD family were engineered to recognize a large variety of restriction sites [32] and used for gene targeting in a wide variety of eukaryotes, including human cells [33], [34]. However, the efficacy of such engineered nucleases is highly variable between the different genomic targets tested [35]. Zinc-finger nucleases (ZFN) were developed for the same purpose. They were built by fusing modular zinc-finger DNA-binding domains to the catalytic domain of the Fok I endonuclease [36]. They were used for targeted gene editing in eukaryotes [37], [38], but seem to induce high toxicity and a high frequency of off-target mutations, probably due to recognition and cutting of many degenerate sequences differing only slightly from the targeted sequence [33]. More recently, a new family of specific endonucleases, called TALEN, was developed. TALENs relie on modular transcription factors, TAL effectors discovered in the genus *Xanthomonas*, a plant pathogen, that can be assembled to recognize any specific DNA sequence [39], [40]. TAL effectors were subsequently fused to Fok I [41], [42] or more recently to Tev I catalytic domains [43], to create modular proteins used in genome editing [44], [45], [46], [47], [48].

In the present work, a TALEN designed to recognize and cut a CAG/CTG trinucleotide repeat was assayed in a dedicated yeast experimental system. We show that, in a diploid strain containing a CAG/CTG trinucleotide repeat integrated in only one of the two homologues, the repeat-containing locus was replaced by its allelic copy by gene conversion, following TALEN induction. In a diploid strain containing CAG/CTG trinucleotide repeats integrated in both homologues, both repeats were shortened by TALEN induction. Deep-sequencing of yeast colonies in which the TALEN was expressed or not expressed showed that induction of the nuclease did not increase the mutation rate, nor did it induce formation of genomic rearrangements, segmental duplications or chromosomal translocations. Therefore, TALENs appear to be the safest and most straightforward way, at the present time, to shorten a trinucleotide repeat to non-pathological lengths.

## Materials and Methods

### Yeast Strains and Plasmids

Both TALEN and split-TALEN were designed and ordered at Cellectis (Paris, France). The target sequence was chosen at the centromeric-proximal junction of the trinucleotide repeat tract. Cellectis validated the design with their own appropriate bioinformatics tools, built the TALEN and validated its cutting efficiency with a dedicated single-strand annealing assay (cleavage rate 0.5 on a scale 0 to 1). Plasmid pCLS9996 (marked with KANMX), carrying the TALEN right arm was transformed in strain GFY40 (*MAT*a *ura3*Δ851 *leu*2Δ1 *his3*Δ200 *lys2*Δ202 *ade2*-opal *SUP4*-opal), or GFY6162-3C (*MAT*a *ura3*Δ851 *leu*2Δ1 *his3*Δ200 *lys2*Δ202 *ade2*-opal *sup4*::CAG), both previously described [25], [26]. Strain GFY6162-3D (*MAT*alpha *ura3*Δ851 *leu*2Δ1 *his3*Δ200 *trp1*Δ63 *ade2*-opal *sup4*::CAG) was transformed with pCLS16715 (TALEN left arm) or with pCLS9984 (split-TALEN left arm), both marked with *LEU2*. Haploid transformants were crossed on rich medium (YPGlucose), and diploids containing both TALEN arms were selected on SC-Leu supplemented with G418 sulfate (200 µg/ml). Repeat lengths were checked by Southern blot in several independent diploids before galactose induction (Figure S1).

### TALEN Induction

Yeast cells were grown overnight in liquid SC -Leu medium supplemented with 200 µg/ml G418 sulfate. Cultures were washed twice with water, diluted to ca. 10^6^ cells/ml and grown in YPLactate for five more hours (one generation). They were diluted to an appropriate concentration, then plated on SC -Leu plates supplemented with 200 µg/ml G418 sulfate, containing either 20 g/l glucose or galactose. Survival was determined as the ratio of CFU on galactose plates over CFU on glucose plates, after 3–5 days of growth at 30°C. Alternatively, after growth in YPLactate, cells were diluted to an appropriate concentration, then plated on SC -Leu -Ade plates supplemented with 200 µg/ml G418 sulfate, containing either 20 g/l glucose or galactose and a small amount of adenine (6.4 mg/l) in order to score colony color. In all induction experiments performed, only a subset of the plated colonies was analyzed by Southern blots and/or PCR.

### Analysis of Trinucleotide Repeat Size

Red and white colonies were picked, total genomic DNA was extracted, digested with Eco RV or Ssp I, loaded on a 1% agarose gel and run overnight at 1 V/cm. The gel was vacuum transfered in alkaline conditions to a Hybond-XL nylon membrane (GE Healthcare) and hybridized with a randomly-labeled probe [49] specific of a unique region downstream of *SUP4*. After washing, the membrane was overnight exposed on a Fujifilm FLA-9000. When repeat tracts were short enough, it was possible to PCR amplify the *SUP4* locus, using primers su3 (TTTCTCGTGTCCCCTCTTCCGT) and su9 (TTCTCTCTGGGTATGTAGGAAT). The PCR fragment was sequenced using a primer (su7: TTCAAGTATTTGTTCATTAATTT) located ca. 210 bp upstream of the repeat tract. Sanger sequencing was performed by GATC Biotech.

### Library Preparation and Deep-sequencing of Yeast Colonies

Each colony, collected on a glucose or on a galactose plate, was grown in non-selective rich medium (YPGlu), whose DNA was extracted and sonicated to an average size of 500 bp (Bioruptor, maximum power (H), 30′′ ON/30′′ OFF cycles, 9 cycles). DNA ends were subsequently repaired with T4 DNA polymerase (15 units, NEBiolabs) and Klenow DNA polymerase (5 units, NEBiolabs) and phosphorylated with T4 DNA kinase (50 units, NEBiolabs). Repaired DNA was purified on two MinElute columns (Qiagen) and eluted in 16 µl (32 µl final for each library). Addition of a 3′ dATP was performed with Klenow DNA polymerase (exo-) (15 units, NEBiolabs). Home-made adapters containing a 4-bp unique tag used for multiplexing, were ligated with 2 µl T4 DNA ligase (NEBiolabs, high concentration, 2×10^6^ units/µl). DNA was size fractionated on a Pippin Prep (Sage Science) and the fraction containing 400–600 bp DNA fragments was recovered in LoBind microtubes (Eppendorf). DNA was PCR amplified with Illumina primers PE1.0 and PE2.0 and Phusion DNA polymerase (1 unit, Thermo Scientific). Depending on PCR efficiency, 9, 12 or 15 PCR cycles were performed on each libary. Twenty-four PCR reactions were pooled, for each library, and purified on Qiagen purification columns (two columns were used for 24 PCR reactions). Elution was performed in 60 µl (twice 30 µl) and DNA was quantified on a spectrophotometer and on an agarose gel.

### Analysis of Paired-end Illumina Reads

One library or two multiplexed libraries were loaded on each lane of a HiSeq 2000 (Illumina), and 110 bp paired-end reads were generated. Reads quality was evalued by FastQC v.0.10.1 (http://www.bioinformatics.babraham.ac.uk/projects/fastqc/) and trimmed off using the paired-end mode of Trimmomatic v0.30 (http://www.usadellab.org/cms/index.php?page=trimmomatic). Trimmed reads were mapped along S288C chromosomes reference sequence (GenBank NC_001133 to NC_001148, PLN 06-DEC-2008), plus the two *SUP4* alleles (*SUP4*-opal and *sup4*-(CAG)) using the paired-end mapping mode of BWA v0.6.2 [50] with default parameters. The output SAM files were converted and sorted to BAM files using SAMtools v0.1.18 [51]. The command *IndelRealigner* from GATK v2.2 [52] was used to realigned the reads. Duplicated reads were removed using the option “*MarkDuplicates”* implemented in Picard v1.81 (http://picard.sourceforge.net/). Reads uniquely mapped to the reference sequence with a minimum mapping quality of 30 (Phred-scaled) were kept. Mpileup files were generated by SAMtools without BAQ adjustments. SNPs and INDELs were called by the options “*mpileup2snp*” and “*mpileup2indel*” of *Varscan2 v2.3.5* [53] with a minimum depth of 5 reads and a threshold of 0.45 for minimum variant allele frequency (strains are diploids). Mismatches were kept when they represented at least 20% of the reads supporting the variant on each strand. They were manually examined and compared between all sequenced libraries for interpretation.

## Results

### TALEN Induction Induces Trinucleotide Repeat Deletions and Contractions

**Figure 1:**
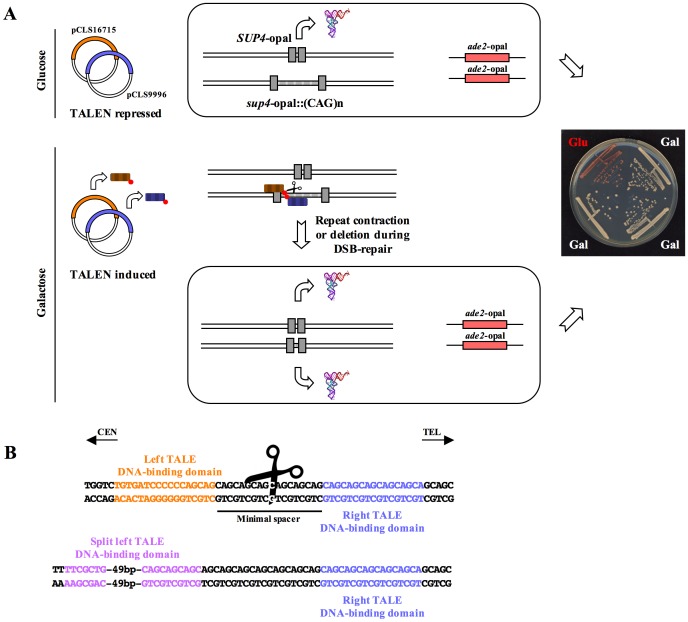
Experimental design. **A**: Plasmids pCLS9996 and pCLS16715, carrying the two TALEN arms, were transformed into *MAT*a and *MAT*alpha haploid strains, and strains were crossed to obtain diploids containing both TALEN arms. The TALEN is normally repressed on glucose medium. One copy of the active *SUP4* tRNA being insufficient to suppress the *ade2*-opal mutation, yeast cells are red [25], [26], [27] (top). In the presence of galactose, the TALEN is expressed, binds CAG/CTG trinucleotide repeats and induces a double-strand break into the repeat tract. If a second copy of an active *SUP4* tRNA is generated during double-strand break repair, the *ade2*-opal mutation will be suppressed and yeast cells will now be white (bottom). **B**: Sequences recognized by both TALE DNA-binding domains and by the split-TALE. The length of the minimal spacer (18 bp) needed to induce a DSB was deduced from repeat tract lengths analyzed in surviving cells after TALEN induction (see text).

**Figure 2:**
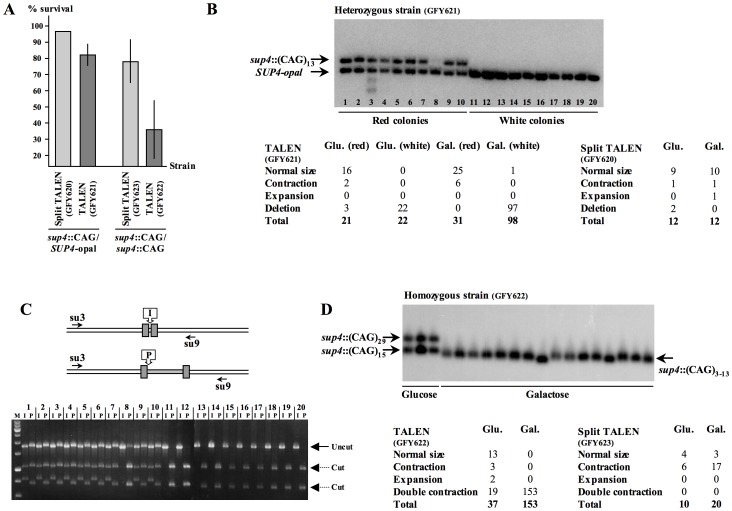
Molecular analysis of survivors after TALEN induction. **A**: Survival after galactose induction. Survival was determined as the ratio of CFU on galactose plates over CFU on glucose plates. GFY620, 621, 622 and 623 are strain names used in these experiments. Error bars indicate 95% confidence intervals. **B**: Molecular analysis of heterozygous diploids (*SUP4*-opal/*sup4*::CAG), on glucose or galactose plates, in strains expressing either the TALEN (GFY621) or the split-TALEN (GFY620). **C**: PCR analysis of heterozygous diploids on glucose or galactose. PCR products were digested using restriction enzyme I-Sce I (I) or Pst I (P). For each clone, numbered 1 to 20, the two lanes show the result of restriction with one of the two enzymes. When both alleles are present, bands of slightly different sizes corresponding to uncut alleles are visible in both lanes (arrow labeled “Uncut”), along with restriction products of cut alleles (arrows labeled “Cut”). When only the *SUP4*-opal allele is present, no cut product is detected in the ‘I’ lane (clones 8 and 11 to 20). Note that these 20 survivors correspond to the same clones in figure 2B (strain GFY621). **D**: Molecular analysis of homozygous diploids (*sup4*::CAG/*sup4*::CAG), on glucose or galactose plates, in strains expressing either the TALEN (GFY622) or the split-TALEN (GFY623). Note that in all induction experiments, only a subset of all colonies growing on glucose or galactose was analyzed by Southern blot and/or PCR.

The genetic assay used in the present work relies on a modified suppressor tRNA gene (*SUP4*) in which the natural intron was replaced by either a short spacer sequence (18 bp, hereafter called *SUP4*-opal) or a CAG/CTG trinucleotide repeat (30–75 triplets, hereafter called *sup4*::CAG). The *SUP4*-opal allele is functional and suppresses an *ade2*-opal non-sense mutation that accumulates a red pigment into yeast cells, whereas the *sup4*::CAG is not functional [25], [26]. Diploid yeast cells carrying homozygous *ade2*-opal mutations are red if only one copy of *SUP4*-opal is present, but they revert to white if two copies are present (Figure 1A). Haploid cells of opposite mating types containing either *SUP4*-opal or *sup4*::CAG, were transformed with one of the two TALEN arms. As a control, a TALEN arm modified to bind a recognition site split in two halves separated by 49 bp, was also transformed in one of the two haploid strains. The left arm of this split-TALEN should not be able to bind its cognate site and therefore no double-strand break should be induced (Figure 1B). TALEN arms are carried by multicopy plasmids (2 micron) and their expression is under the control of the inducible *GAL1-10* promoter [54], [55]. Cells were simultaneously plated on glucose and galactose media and colonies were scored after 3–5 days of growth. Yeast survival to the TALEN induction was 81.4%±7.2%, slightly less than survival to the split-TALEN induction (96.4%, Figure 2A). White colonies were scored and represent a majority of cells on both media, even though they are more frequent on galactose (82.5% of white colonies) as compared to glucose (66.7%). This suggests that even in repressing conditions (glucose), the *GAL1-10* promoter shows some level of leakiness which is, associated to multicopy plasmids, apparently sufficient to induce TALEN expression. In support of this observation, we noticed that when crossing two haploids strains containing a stable trinucleotide repeat and one of the two TALEN arms, none of the diploids obtained contained a repeat longer than 30 triplets, strongly suggesting than even in repressing conditions, leaky expression of both TALEN arms occur to a level high enough to induce repeat contractions when both plasmids are in the same diploid cell (Figure S1). Quantification of steady-state TALEN transcripts in glucose and galactose media confirmed a low but detectable level of transcripts in glucose (Figure S2).

DNA originating from red and white colonies was subsequently analyzed by Southern blotting. Forty-nine out of 52 red colonies contain the two alleles, only three colonies showed the complete deletion of the *sup4*::CAG allele (Figure 2B). Conversely, 119 out of 120 white colonies only contain the *SUP4*-opal allele, whose signal intensity was twice the intensity detected in red colonies, suggesting that it corresponds to a near-complete deletion of the *sup4*::CAG allele. We took advantage of a restriction site polymorphism between *SUP4*-opal and *sup4*::CAG alleles, to discriminate between a perfect homozygotization and a large contraction of the *sup4*::CAG allele. DNA extracted from red or white diploid survivors was amplified and digested with enzymes recognizing one of the two alleles. In all ten white survivors analyzed, restrictions showed the presence of only the *SUP4*-opal allele (Figure 2C). Sequencing the same PCR products amplified from white diploid survivors confirmed that only one sequence was present, and not a mix of two different sequences, as would be expected for an heterozygous *SUP4*/*sup4* locus. These experiments proved that gene conversion of the *sup4*::CAG allele by the *SUP4*-opal allele was more than 99% efficient following TALEN expression. Comparatively, there was no difference between glucose and galactose and no gene conversion was detected when inducing the split-TALEN (Figure 2B).

In a second set of experiments, we built a diploid strain containing two *sup4*::CAG alleles of different lengths. In such a strain, it is not possible to screen for white colonies, since both alleles are deficient in suppressing *ade2*-opal mutation. In the diploid strain containing the split-TALEN (GFY623), survival to galactose induction was 78.1%±13.7%, a slightly lower figure than survival of the *SUP4*-opal/*sup4*::CAG heterozygote (GFY620, 96.4%, Figure 2A). However, in the diploid strain containing the TALEN (GFY622), survival dropped to 37.1%±18%, a figure 2.2 fold lower than survival of the *SUP4*-opal/*sup4*::CAG heterozygote. This shows that cutting both chromosomes instead of one decreases viability by about a two-fold factor. Molecular analysis showed that ca. 5% of colonies on glucose (2 out of 37) showed a small expansion, whereas 59% (22 out of 37) of colonies exhibited a contracted or deleted allele (Figure 2D), suggesting again that some TALEN induction occurs in repressing conditions.

**Figure 3:**
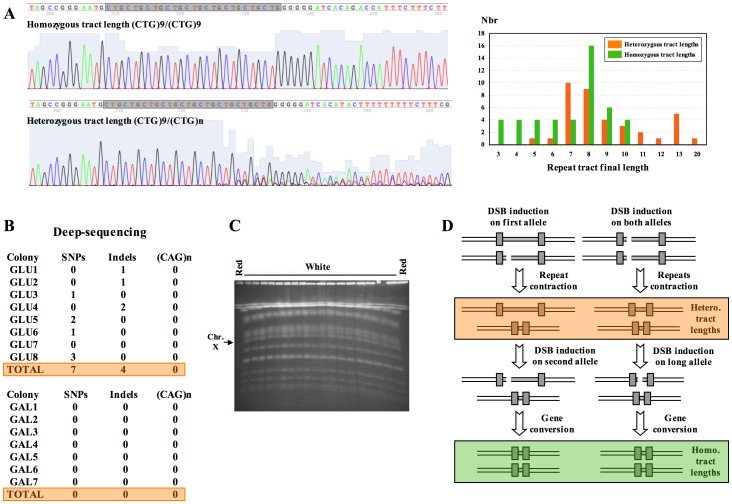
Karyotypes and sequencing of TALEN-induced yeast colonies. **A**: Sanger sequencing of survivors. PCR fragment amplified with su3/su9 (Figure 2C) was sequenced using a primer (su7) located ca. 210 bp upstream of the repeat tract. Left: when only one allele was present, one unique sequence was read (upper graph, homozygous tract length). When two alleles of different lengths were present, the sequence was blurry and unreadable after the shortest of the two repeat tracts (lower graph, heterozygous tract length). The freeware 4Peaks was used to visualize sequences. Right: length distribution of alleles in homozygous tract length (green bars) and heterozygous tract length (orange bars) survivors to TALEN induction. Homozygous tract lengths are shorter on the average (mean = 7 triplets) than heterozygous tract lengths (mean = 9 triplets), this difference being very significant (Wilcoxon test, p-value = 0.0021). Note that for heterozygous alleles only the length of the shortest repeat can be precisely known, hence the statistical difference observed between the two distributions is more significant than shown. **B**: Deep sequencing of yeast genomes from yeast colonies isolated on glucose or galactose plates. Each of the 15 yeast genomes was resequenced to 700 X coverage, on the average (see Table S1). For each colony, the number of unique SNPs, insertions/deletions or size changes in other CAG/CTG triplet repeats of the yeast genome, are indicated. **C**: Pulse-field gel electrophoresis of red and white colonies after galactose induction. Chromosomal DNA was prepared from yeast cells embedded in agarose plugs according to standard methods [72]. Agarose plugs were loaded on 1% agarose gels (SeaKem GTG, TEBU) and electrophoresis was run on Rotaphor (Biometra) at 12°C in 0.25X TBE buffer at pH8.3, 140V, with decreasing pulse ramp 140 sec to 80 sec, and field angle 120°. Karyotypes are identical among all clones and do not show any large chromosomal rearrangement, neither on chromosome X (bearing *SUP4*) nor on any other chromosome. **D**: Two models proposing how heterozygous and homozygous tract lengths may be formed following TALEN induction (see text).

In galactose, 100% of the 153 colonies analyzed showed one single band corresponding in size to the near-complete contraction of both repeat tracts (Figure 2D: Double contraction). However, Southern blot resolution was not sufficient to determine if both alleles harbored repeats of the exact same length. DNA extracted from diploid survivors was therefore amplified and sequenced. In 23 out of 60 sequenced survivors (38%), only one sequence was present, as shown by good quality, evenly spaced peaks (Figure 3A). In 37 out of 60 survivors (62%), a mix of two DNA sequences was read after the repeat tract, indicating that the two alleles carry repeat tracts of different lengths. Using this approach, only the shortest of the two repeat tract lengths could be determined, and was found to range from three to 13 triplets (with one exception, one sequence of 20 triplets was found). Therefore, the minimal spacing between the two TALE DNA-binding domains necessary to obtain active dimerization of the Fok I nuclease and subsequent DSB formation was calculated as being 39 nt (13 triplets) minus the number of triplet repeat nucleotides bound to the left TALE (5 nt) and the number of triplet repeat nucleotides bound to the right TALE (16 nt, Figure 1B), for a total of 18 nt, a figure slightly higher than expected but compatible with former reports [56].

When survivors of the *sup4*::CAG/*sup4*::CAG strain containing the split-TALEN were analyzed, no colony containing two contracted alleles was detected (Figure 2D). However, six colonies out of ten on glucose and 17 colonies out of 20 on galactose showed a contraction of the largest of the two alleles (scored as “Contraction”, Figure 2D). This strongly suggests that presence of the right TALE DNA-binding domain is sufficient to increase the instability of a CAG trinucleotide repeat, probably by interfering with repeat tract replication. Indeed, when only the right TALE DNA-binding domain was expressed in a haploid strain containing a *sup4*::CAG allele, stability of the repeat tract was decreased (our own unpublished data).

### TALEN Induction does not Increase Mutation Rates

In order to determine TALEN specificity, particularly if an increase in off-site mutations was associated with its expression, we completely resequenced eight colonies growing on glucose plates and seven colonies growing on galactose plates. Paired-end Illumina reads were generated and mapped to the S288C reference genome for each colony (Table S1). After removal of duplicates, coverage of unique sequences was homogeneous in all 15 clones sequenced, showing no aneuploidy nor segmental duplication. Among eight glucose colonies, seven unique heterozygous SNPs were detected, whereas among seven galactose colonies no heterozygous SNP was detected (Figure 3B). These numbers are not significantly different from each other and are in good agreement with predictions. Lynch and colleagues [57] determined that the average base substitution rate per nucleotide site was 3.3×10^−10^ per cell division, in *S. cerevisiae.* Given that glucose and galactose colonies underwent respectively 33 and 30 cell divisions before DNA was extracted and sequenced, following a Poisson distribution it was expected that ca. 11% of the sequenced colonies (one or two colonies out of 15) contained at least one mutation. Four colonies out of 15 (27%) actually contained at least one mutation, a number not statistically different from expected (Fisher exact test p-value: 0.43 for two mutant colonies, 0.21 for one mutant colony).

Mutant colonies contained between one (two colonies) and three SNPs (one colony). Altogether, three transitions for four transversions were found, a proportion not statistically different from expected (expected ratio: 0.61 [57]). Insertions and deletions (Indels) of one base pair in non-monotonous DNA are expected to be ten times less frequent than base substitutions [58], whereas indels within long poly-A/T stretches (12 bp) are more frequent [57]. Indeed, we only found one deletion of a GC dinucleotide in an intergenic region (zero expected) and three colonies containing indels in monotonous poly-A/T stretches (four expected). More importantly no mutation was detected in any one of the naturally occuring 88 CAG/CTG trinucleotide repeats (at least five triplet long ) of the S288C genome [59]. All indels and one out of seven SNPs fall within intergenic regions. Out of six remaining SNPs in coding regions, two are synonymous (third codon base) whereas four are non synonymous and encode point mutations in five different genes (Table S2). We concluded that expression of a TALEN targeted to a specific CAG/CTG trinucleotide repeat has no effect on other triplet repeats nor on the overall mutation rate of the yeast genome.

Since deep-sequencing cannot reveal reciprocal translocations that could be induced by the TALEN, as a last control experiment, a PFGE was run on the heterozygous *SUP4*-opal/*sup4*::CAG strain. DNA from two colonies grown on glucose and 20 colonies grown on galactose was prepared embedded in agarose plugs and loaded on a PFGE. All karyotypes were normal, showing no evidence for aneuploidies, large segmental duplications or translocations (Figure 3C).

## Discussion

In the present work, we show that a TALEN designed to recognize and cut a CAG/CTG trinucleotide repeat integrated in a yeast chromosome was 100% efficient in shortening the repeat tract, without inducing any other mutation in the yeast genome. In a former similar experiment, a zinc-finger nuclease was designed to recognize and cut a plasmid-born CAG/CTG trinucleotide repeat tract, in human cells. It was shown to increase triplet repeat instability by 15 fold, inducing repeat contractions, deletions of the complete repeat tract along with flanking DNA sequences, and insertions of plasmidic DNA within the repeat tract [60]. In other experiments, in which a ZFN was directed at chromosome-borne CAG/CTG repeats in human cells [61], frequent contractions were observed, along with less frequent expansions of the repeat tract. These expansions were proposed to occur by homologous recombination between sister chromatids in S or G2 phase of the cell cycle. However, in these two previous experiments with ZFN, no estimate of the rate of genome wide off-target mutations induced by the nuclease was provided. Targeting efficiencies between ZFN and TALEN have been compared in nematode [62] and drosophila [63]. Both ZFN and TALEN are mutagenic, making a double-strand break that will be repaired by unfaithful non-homologous end joining, generating mutations at the broken locus. However, frequencies of mutagenesis vary greatly between ZFN and TALEN, depending on the locus targeted. It is possible that TALEN efficacy, as compared to ZFN, at recognizing and cutting CAG/CTG secondary structures, is due to different DNA-protein structures. TAL Effectors wrap around DNA, each repeated motif consisting of two alpha helices connected by a short loop containing the Repeat Variable Diresidue (RVD). Each RVD contacts its cognate nucleotide within the major groove, and the protein is wrapped around DNA in a superhelical structure [64]. Therefore, if some secondary structures are formed within triplet repeats, it is possible that they are disrupted by the binding of the TALEN left arm at the repeat junction (Figure 1B), allowing efficient binding of the TALEN right arm on the triplet repeat sequence. Very recently, a new family of highly specific endonucleases, called CRISPR, based on a guide RNA associated to a bacterial nuclease (Cas9), was engineered to modify eukaryotic genomes. The guide RNA is homologous to the target sequence and only 20–30 base pairs of homology are required for the RNA-nuclease complex to recognize and cut its cognate sequence [65]. Given that CUG-containing RNAs are known to form stable secondary structures [66], it is probable that these structures will interfere either with binding of the RNA guide to the nuclease or recognition of the cognate sequence, making it unlikely that CRISPR will be very efficient at recognizing and cutting structured sequences. More importantly, the CRISPR-associated protein (Cas9) requires a protospacer adjacent motif (PAM) whose sequence is 5′-NGG-3′, in order to bind and cleave its target [67]. Since there is no NGG triplet in a CAG/CTG trinucleotide repeat, at the present time the CRISPR technology cannot be used to induce a DSB into such repeats.

It will be interesting to test the efficacy and specificity of a similar TALEN in mammalian cells containing large CAG/CTG trinucleotide repeat expansions. The DM1 locus, containing such an expansion in myotonic dystrophy patients has been well studied by several authors. It was shown that the triplet repeat instability at this locus was dependent on the presence and activity of a nearby CTCF binding site [68]. Given that CTCF is a regulatory factor involved in chromatin remodeling, and that it plays a direct role in regulating replication (and therefore DNA accessibility) at the DM1 locus [69], its presence could affect the efficacy of recognition and binding of the trinucleotide repeat by the TALEN. Additional experiments will be needed to properly adress these questions.

The mechanism by which CAG/CTG trinucleotide repeats are shortened by the TALEN can only be infered from former experiments with I-Sce I [26] and from known pathways of DSB-repair in yeast [70]. DSBs made in heterozygous diploids are repaired almost exclusively by gene conversion, effectively removing the repeat tract (Figure 2B). However, six cases of repeat contractions (in red colonies) were detected on galactose plates. These contractions may correspond to intramolecular repair of the DSB by single-strand annealing, leading to small shortenings of the repeat tract. Alternatively, it may also correspond to natural instability of the trinucleotide repeat. In diploids homozygous for repeat tracts, it is likely that DSBs are repaired by single-strand annealing, although this must be confirmed by redoing similar experiments in dedicated yeast mutants. Homozygous survivors may result from iterative coordinated or uncoordinated breaks on both chromosomes, one (or two) allele(s) being cut and repaired by intra-molecular mechanism, while the other allele is repaired by gene conversion using the shortest one as a template (Figure 3D). Heterozygous survivors may result as before, from iterative coordinated or uncoordinated breaks, that will not be repaired by gene conversion and will therefore lead to repeat tracts of different lengths. This may be due to the presence of CAG repeats at DSB ends, which may impede one or more steps of homologous recombination, including correct processing of the break, subsequent formation of Rad51 nucleofilament, or strand invasion of the homologous template (which also contains CAG repeats). In support of this hypothesis, distribution of repeat tract lengths among heterozygous and homozygous survivors shows that homozygous tract lengths are shorter on the average (mean = 7 triplets) than heterozygous tract lengths (mean = 9 triplets), this difference being very significant (Wilcoxon test, p-value = 0.0021, Figure 3A). This suggests that gene conversion between repeat tracts may be hindered when tract lengths are too long, probably inhibiting an early step in the recombination process. In these cases, intramolecular repair is favored, giving rise to longer repeat tracts of unequal lengths. However, we cannot totally exclude that heterozygous survivors result from slippage occuring during DNA synthesis associated to gene conversion, as was previously demonstrated for CAG/CTG trinucleotide repeats [25], [26], [27]. In our previous work, when an I-Sce I DSB was induced between two short (CAG)_5_ tracts, the break was repaired by annealing between the two repeats 67% of the time [26], although a homologous donor sequence was also available, a figure close to the proportion of heterozygous survivors obtained here. This suggests that when competition is possible between intra- and intermolecular repair mechanisms, intramolecular events are favored, even though gene conversion is highly efficient in yeast [70]. Interestingly, it was very recently shown that induction of a TALEN, 129 bp downstream of a (TG)_70_ dinucleotide repeat in zebrafish induced frequent contractions of the repeat tract [71]. In this experiment, three types of mutations were obtained: 56% of the sequenced zebrafish embryos showed a contraction of the TG tract but no modification of the TALEN recognition site, 15% of the embryos exhibited mutations of the recognition site but an unchanged TG tract, and 5% of the embryos showed both modifications of the recognition site and repeat contraction. Various modes of DSB-repair are proposed to account for mechanism(s) contracting tandem repeats 129 bp away from a DSB. It would be interesting to know whether mechanisms involved in zebrafish to repair TALEN-induced DSBs are similar to those happening in yeast.

TALEN expression leads to trinucleotide repeat contractions with a 100% efficacy in yeast cells, giving rise to survivors containing homozygous or heterozygous shorter alleles. Although precise molecular mechanisms by which contractions occur following TALEN induction may only be infered from our knowledge of DSB-repair following irradiation, drugs or meganuclease action, yeast will certainly prove to be helpful in dissecting mechanisms of trinucleotide repeat contractions induced by a TALEN.

## Supporting Information

Figure S1 **Instability of trinucleotide repeats in diploid strains containing TALEN or split-TALEN, on glucose medium.** A: Left: strains GFY6161-3C (*MAT*a *leu2*Δ1 *his3*Δ200 *lys*2Δ202 *ade2*-opal *sup4*::(CAG)_30_) and GFY6162-3D (*MAT*alpha *ura3*Δ851 *leu*2Δ1 *his3*Δ200 *trp1*Δ65 *ade2*-opal *sup4*::(CAG)_75_) were respectively transformed with pCLS9996 (KANMX marker) or pCLS16715 (*LEU2* marker). Six transformants were analyzed by Southern blot, for each strain, to estimate repeat length variability after transformation, as well as the untransformed strain as a size control (labeled “C”). On each gel a ladder corresponding in size to different triplet repeat lengths, hybridizing with the probe, was loaded in the rightmost lane. Transformant #3 in strain GFY6162-3C shows extensive contractions of the repeat tract, but all other transformants exhibit stable trinucleotide repeats after transformation. Right: Transformants GFY6162-3C/1 and GFY6162-3D/1 were crossed, and diploids were selected on glucose SC-Leu plates supplemented with G418 sulfate (200 µg/ml). Twelve independent diploids were analyzed by Southern blot, as previously. None of the diploids contained the repeat band around 75 triplets, showing that it was contracted during or right after the cross, even though cells were crossed on glucose medium. In this particular cross, diploid #5 was selected for further induction experiments. B: Left: strain GFY6162-3D (*MAT*alpha *ura3*Δ851 *leu*2Δ1 *his3*Δ200 *trp1*Δ65 *ade2*-opal *sup4*::(CAG)_75_) was transformed with pCLS9984 (split-TALEN left arm) and 12 independant transformants were analyzed by Southern blot. Transformant #3 shows an expansion and transformant #6 shows a contraction of the repeat tract, but all other transformants exhibit stable trinucleotide repeats after transformation. Clone GFY6162-3D/2 was crossed to GFY6162-3C/1, and diploids were selected on glucose SC-Leu plates supplemented with G418 sulfate (200 µg/ml). Right: Molecular analysis of four diploids shows that two of them (#3 and #4) exhibit a large contraction of the (CAG)_75_ repeat, that occured during or right after the cross, even though cells were crossed on glucose medium and the split-TALEN is not active. The two haploid parental strains are used as an additional size control (labeled “C”). In this particular cross, diploids #3 and #4 were selected for further induction experiments of the split-TALEN.
(PDF)

Figure S2 **Steady-state level of TALEN transcript in repressing (glucose) and inducing (galactose) conditions.** Strains GFY621 (*SUP4*-opal/*sup4*::CAG) and GFY622 (*sup4*::CAG/*sup4*::CAG) were grown overnight in liquid SC -Leu glucose medium supplemented with 200 µg/ml G418 sulfate. Cultures were washed twice with water, diluted to ca. 10^7^ cells/ml and grown in 20 ml SC -Leu glucose or galactose medium supplemented with 200 µg/ml G418 sulfate, for four hours. Total RNAs were extracted and analyzed by Northern blot as previously described [73], [74]. The full left TALE arm purified from pCLS16715 was labeled by random priming and used as probe. Blots were stripped in boiling 0.5% SDS and rehybridized with a randomly labeled actin probe, covering the *ACT1* yeast gene. Membranes were exposed and signals were quantified on a Fujifilm FLA-9000. Relative amounts of TALEN as compared to actin transcripts are shown on the graph, in both growth conditions. There is a 10–32 fold increase of TALEN transcripts in galactose as compared to glucose, depending of the strain. In GFY622 the level of TALEN transcripts is lower than in GFY621, in both conditions. The reason for this difference was not further investigated. Note that the level of actin is lower in galactose as compared to glucose, reflecting that cells in galactose grew more slowly than in glucose medium, reducing the final number of cells, and therefore the final amount of RNAs extracted in glucose as compared to galactose.
(PDF)

Table S1 **Illumina sequencing data.** Each library corresponds to one individual colony, collected on glucose or galactose plates (Origin). Total number of reads, initial read lengths, lengths after trimming and sequencing depths are indicated for each sequenced library.
(PDF)

Table S2 **Summary of mutations detected in the 15 sequenced colonies.**
(PDF)
