# Supplementary figures and images for: Highly Specific Contractions of a Single CAG/CTG Trinucleotide Repeat by TALEN in Yeast
Source: PLoS One. 2014 Apr 18;9(4):e95611. doi: 10.1371/journal.pone.0095611 (PMC3991675; doi:10.1371/journal.pone.0095611)

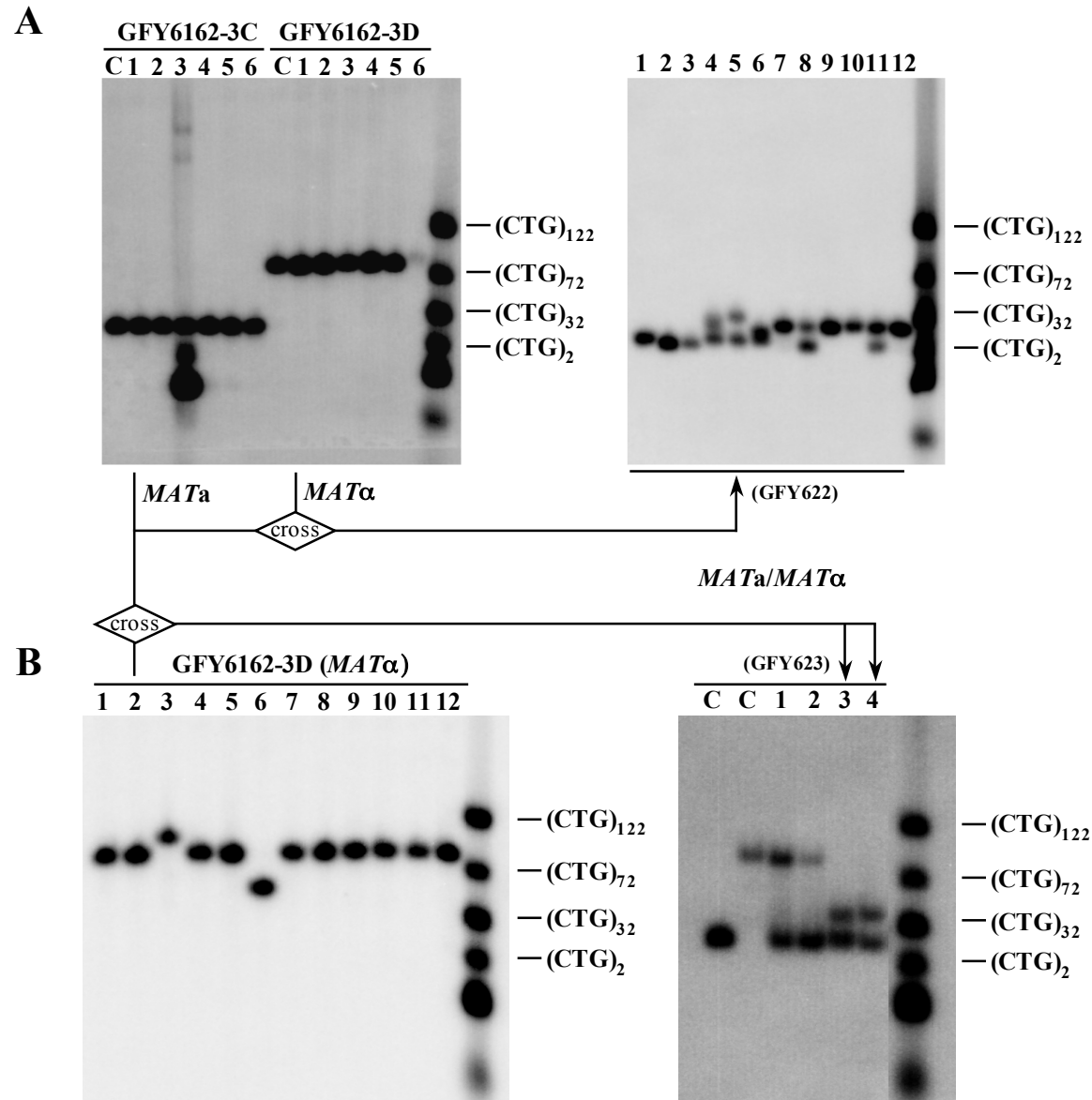

Supplement: Figure S1 — Instability of trinucleotide repeats in diploid strains containing TALEN or split-TALEN, on glucose medium. A: Left: strains GFY6161-3C (MATa leu2Δ1 his3Δ200 lys2Δ202 ade2-opal sup4::(CAG)30) and GFY6162-3D (MATalpha ura3Δ851 leu2Δ1 his3Δ200 trp1Δ65 ade2-opal sup4::(CAG)75) were respectively transformed with pCLS9996 (KANMX marker) or pCLS16715 (LEU2 marker). Six transformants were analyzed by Southern blot, for each strain, to estimate repeat length variability after transformation, as well as the untransformed strain as a size control (labeled “C”). On each gel a ladder corresponding in size to different triplet repeat lengths, hybridizing with the probe, was loaded in the rightmost lane. Transformant #3 in strain GFY6162-3C shows extensive contractions of the repeat tract, but all other transformants exhibit stable trinucleotide repeats after transformation. Right: Transformants GFY6162-3C/1 and GFY6162-3D/1 were crossed, and diploids were selected on glucose SC-Leu plates supplemented with G418 sulfate (200 µg/ml). Twelve independent diploids were analyzed by Southern blot, as previously. None of the diploids contained the repeat band around 75 triplets, showing that it was contracted during or right after the cross, even though cells were crossed on glucose medium. In this particular cross, diploid #5 was selected for further induction experiments. B: Left: strain GFY6162-3D (MATalpha ura3Δ851 leu2Δ1 his3Δ200 trp1Δ65 ade2-opal sup4::(CAG)75) was transformed with pCLS9984 (split-TALEN left arm) and 12 independant transformants were analyzed by Southern blot. Transformant #3 shows an expansion and transformant #6 shows a contraction of the repeat tract, but all other transformants exhibit stable trinucleotide repeats after transformation. Clone GFY6162-3D/2 was crossed to GFY6162-3C/1, and diploids were selected on glucose SC-Leu plates supplemented with G418 sulfate (200 µg/ml). Right: Molecular analysis of four diploids shows that two of them (#3 and #4) exhibi [file pone.0095611.s001.pdf]

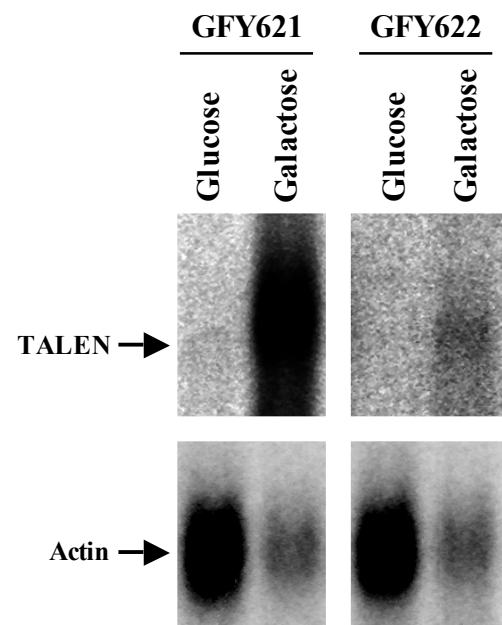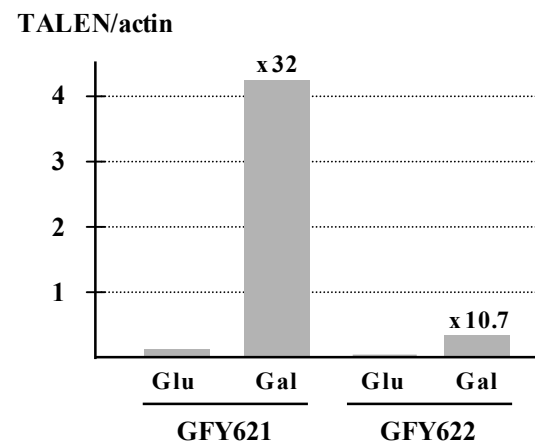

Richard *et al.*  
Supporting Figure S2

Supplement: Figure S2 — Steady-state level of TALEN transcript in repressing (glucose) and inducing (galactose) conditions. Strains GFY621 (SUP4-opal/sup4::CAG) and GFY622 (sup4::CAG/sup4::CAG) were grown overnight in liquid SC -Leu glucose medium supplemented with 200 µg/ml G418 sulfate. Cultures were washed twice with water, diluted to ca. 107 cells/ml and grown in 20 ml SC -Leu glucose or galactose medium supplemented with 200 µg/ml G418 sulfate, for four hours. Total RNAs were extracted and analyzed by Northern blot as previously described [73], [74]. The full left TALE arm purified from pCLS16715 was labeled by random priming and used as probe. Blots were stripped in boiling 0.5% SDS and rehybridized with a randomly labeled actin probe, covering the ACT1 yeast gene. Membranes were exposed and signals were quantified on a Fujifilm FLA-9000. Relative amounts of TALEN as compared to actin transcripts are shown on the graph, in both growth conditions. There is a 10–32 fold increase of TALEN transcripts in galactose as compared to glucose, depending of the strain. In GFY622 the level of TALEN transcripts is lower than in GFY621, in both conditions. The reason for this difference was not further investigated. Note that the level of actin is lower in galactose as compared to glucose, reflecting that cells in galactose grew more slowly than in glucose medium, reducing the final number of cells, and therefore the final amount of RNAs extracted in glucose as compared to galactose. (PDF) [file pone.0095611.s002.pdf]
