# Supplementary material for: Highly Specific Contractions of a Single CAG/CTG Trinucleotide Repeat by TALEN in Yeast
Source: PLoS One. 2014 Apr 18;9(4):e95611. doi: 10.1371/journal.pone.0095611 (PMC3991675; doi:10.1371/journal.pone.0095611)
Supplement: Table S1 — Illumina sequencing data. Each library corresponds to one individual colony, collected on glucose or galactose plates (Origin). Total number of reads, initial read lengths, lengths after trimming and sequencing depths are indicated for each sequenced library. (PDF) [file pone.0095611.s003.pdf]

Supporting Table S1: Illumina sequencing data

Origin	Library	Total reads	Initial read length (bp)	Read length after trimming (bp)	Median sequencing depth
Galactose	GAL1	298 x 10^6	110	82	1601 X
	GAL2	119.6 x 10^6	110	82	677 X
	GAL3	134.4 x 10^6	110	82	780 X
	GAL4	117.8 x 10^6	110	82	675 X
	GAL5	262.2 x 10^6	110	82	765 X
	GAL6	167.6 x 10^6	110	82	975 X
	GAL7	155.4 x 10^6	110	82	1779 x
Glucose	GLU1	41.2 x 10^6	110	83	457 X
	GLU2	41.2 x 10^6	110	83	457 x
	GLU3	70 x 10^6	110	83	394 X
	GLU4	118 x 10^6	110	83	648 X
	GLU5	54 x 10^6	110	83	303 X
	GLU6	28 x 10^6	110	83	156 X
	GLU7	44 x 10^6	110	83	249 X
	GLU8	100 x 10^6	110	83	588 X

Each library corresponds to one individual colony, collected on glucose or galactose plates (Origin). Total number of reads, initial read lengths, lengths after trimming and sequencing depths are indicated for each sequenced library.
