# Supplementary material for: Highly Specific Contractions of a Single CAG/CTG Trinucleotide Repeat by TALEN in Yeast
Source: PLoS One. 2014 Apr 18;9(4):e95611. doi: 10.1371/journal.pone.0095611 (PMC3991675; doi:10.1371/journal.pone.0095611)
Supplement: Table S2 — Summary of mutations detected in the 15 sequenced colonies. (PDF) [file pone.0095611.s004.pdf]

Supporting Table S2: summary of mutations detected in the 15 sequenced colonies

Base substitutions:

**Chromosome**	**Position [1]**	**Mutation**	**Location**	**Codon**	**Amino acid**
III	300201	C->A	Intergene	-	-
IV	628439	C->A	*RLI1*	GTG->TTG	Val->Leu
IV	1298899	G->T	*SYF1*	GTT->TTT	Val->Phe
X	333003	A->T	*ZAP1*	ACT->ACA	Synonymous
X	626414	T->C	*ECM27*	TTT->CTT	Phe->Leu
XI	142750	C->T	*PIR1*	CCG->CCA	Synonymous
XV	1075334	A->G	*YOR389w*	AAC->AGC	Asn->Ser

Insertions/deletions:

**Chromosome**	**Position [1]**	**Mutation**	**Sequence**	**Location**
II	809788	-T	$(T)_{19}$	Intergene
VI	106271	+TT	$(T)_{13}$	Intergene
VII	95081	-GC	Non monotonous	Intergene
VII	413969	-GA	$(A)_2G(A)_{12}$	Intergene

[1] mutation position according to GenBank NC_001133 to NC_001148, PLN 06-DEC-2008 yeast genome assembly
